# A Little Shock for Tobacco Smokers

**Published:** 1920-11-06

**Authors:** 


### A Little Shock for Tobacco Smokers.

It is assumed by ninety-nine smokers out of a hundred, without any particular justification beyond the known fact that tobacco smoke disagrees with the comfort of the green-fly, that if they smoke with consistent regularity they render themselves relatively safe from disease. During the influenza epidemics especially it is a highly popular and soothing theory that there is wisdom in this advice:

> Let those now smoke who never smoked before,
> And those who always smoked now smoke the more.

But the *Lancet* has been attempting to shake the faith of enthusiasts by pointing out that we cannot be so sure, after all. The smoke problem is a real one, to which no very definite answer seems to have been given. Tobacco certainly has a destructive effect on germs that have been prepared for laboratory experiments, but the conditions are rather different when the microbes have established themselves in the mouths of pipe- and cigarette-smokers. An Italian professor has, however, taken the inquiry a stage further by experimenting on the effect of tobacco smoke in the human mouth. It is a little discouraging to find his conclusion to be that "a bacteriological action was only shown to follow the consumption of very large quantities of tobacco, and then only on micro-organisms of least resistance, such as the meningococcus and cholera vibrio." In other words, comments an apparently disillusioned writer in the *Manchester Guardian*, the smoker would have to smoke very hard and very long, and then only interfere with the weaklings of the hostile force. There is, however, another side to the matter—what may be termed the spiritual as opposed to the physical side. As long as the prodigious smoker firmly believed that by prodigious smoking he was warding off an attack of influenza during a severe epidemic he was undoubtedly putting himself in a frame of mind calculated to give him a greater resisting power against the onset of the disease. If the scientific truth of the matter, generally disseminated by non-smokers who hate tobacco, causes him to lose his faith, he will be less psychologically immune. But, after all, he may do well to continue to smoke hygienically—as long as he can avoid smoker's heart. The theory that he loves has not yet been incontestably disproved, and at any rate he is reasonably safe from the meningococcus and cholera vibrio.

